# Assessment of left ventricular systolic function using two- and three-dimensional speckle tracking echocardiography among healthy preschool-age pediatric children

**DOI:** 10.1186/s43044-022-00258-w

**Published:** 2022-03-28

**Authors:** Heba Kamel, Ayah Tarek Elsayegh, Hany Nazmi, Hebatallah Mohamed Attia

**Affiliations:** Congenital and Structural Heart Disease Unit, Cardiology Department, Ain Shams University Hospital, Nargess 3, Fifth Settlement, Abbassya, Cairo, 11835 Egypt

**Keywords:** Two-dimensional strain echocardiography, Three-dimensional strain echocardiography, Left ventricle functions

## Abstract

**Background:**

Accurate measurements of left ventricular (LV) volumes and function are important in the management of patients with various cardiac abnormalities. Two-dimensional (2D) speckle tracking echocardiography (STE) is shown to be accurate in detecting subclinical myocardial dysfunction when most of the conventional echocardiography parameters were normal. Three-dimensional (3D) echocardiography is a new noninvasive imaging technique that has been shown to be accurate in determining cardiac volume and performance. Establishment of normal range values of 3D STE over a different range of ages is crucial before applying this recent technology in clinical applications. This study aimed to assess feasibility of 3D LV STE and establish normal values for the LV systolic function among healthy Egyptian preschool-age pediatric population using 2D and 3D STE.

**Results:**

A total of 200 subjects (95%) met the criteria for 2DSTE analysis, 10 were excluded from the 2D analysis and 180 subjects (85%) met the criteria for 3D STE analysis. Regarding the 2D STE GLS, the mean was -22.1345 ± 2.166%, GCS was f -19.02 ± 1.23%, and GRS was 42.25 ± 2.35%. There was a strongly positive significant correlation between age and 2D values of GLS (*P* = 0.001). The GCS showed a weakly positive nonsignificant correlation with age (*P* = 0.28), while GRS showed a strongly negative significant correlation with age (*P* = 0.001). Regarding the 3D STE data, GLS mean was -20.48 ± 1.526%, GCS mean was -13.90 ± 2.05%, while GRS mean was 47.21 ± 2.382%. 3D GLS values had a strongly positive significant correlation with age (*P* = 0.001). While GCS showed a weakly positive nonsignificant correlation (*P* = 0.955), GRS showed a strongly negative significant correlation (*P* = 0.001). Linear correlation analysis of 2D and 3D values of strain showed that GLS had a strongly positive significant correlation (*P* = 0.001), while GCS showed a weakly positive nonsignificant correlation (*P* = 0.161) and GRS showed a strongly positive significant correlation (*P* = 0.001).

**Conclusions**

3D global strain analysis using the 3D STE is feasible in the preschool-age pediatric population. Results were almost concordant with previous observations in most of the values except for GCS, especially 3D values which could be attributed to different vendor system used and different ethnicity. Further studies are required to reinforce these data using the GE vendor machine.

## Background

Accurate measurements of left ventricular (LV) volumes and function are important in the management of patients with various cardiac abnormalities. In the past years, studies have shown that assessment of the LV systolic function is both important for diagnosis and prognosis and crucial for management of various clinical scenarios [1]. The usual parameters used in practice, such as ejection fraction (EF) by motion (M) mode, velocities by pulsed-wave (PW) tissue Doppler imaging (TDI), give valuable information on global myocardial function but with limitations [2].

Myocardial deformation by two-dimensional (2D) speckle tracking echocardiography (STE) partly bypasses the limitations. STE could be a relatively reproducible technique which is also independent of angle of insonation and provides assessment of both global and regional myocardial systolic and diastolic function in several important situations [2]. Also, it is shown to be accurate in detecting subclinical myocardial dysfunction when most of the conventional echocardiography parameters were normal or reported inconsistent results [3].

Yet also due to some limitations of 2D STE for the assessment of LV mechanics, recent investigations have redirected to STE analysis by real-time three-dimensional echocardiographic (RT3DE) data [4]. Three-dimensional echocardiography (3DE) is a new noninvasive imaging technique that has been shown to be accurate in determining cardiac volume, mass, and performance [5].

The myocardium of the LV has three different myocardial layers, and they contract in different directions at the same time, so precise assessment necessitates three-dimensional (3D) analysis. 3DE different vendor machines with numerous STE software’s enabled us to perform clinical 3D strain through the past years [6].

Establishment of normal range values of 3D STE over a different range of ages is crucial before applying this recent technology in clinical applications as detecting subclinical LV dysfunction in subjects with normal LV EF, also assessing the affection of the LV function with different drug therapy applications, evaluate LV mechanics during regular follow-ups. In addition to that, also previous study has detected age-dependent differences in a wide range of LV functions studied by TDI or 2D STE. Therefore, the age dependency of 3D strain parameters should also be assessed [4]. So, this study aimed to assess feasibility of 3D LV speckle analysis and establish normal values for the LV systolic function among healthy Egyptian preschool-age pediatric population using 2D and 3D STE.

## Methods

### Study population

From February 2021 to August 2021, a total of 210 consecutive subjects from birth to 6 years of age were prospectively enrolled for elective echocardiography assessment in our congenital and structural heart disease unit, Cardiology Department, Faculty of Medicine, X University. The inclusion criteria were (a) referral for echocardiographic assessment for clinical causes, (b) no evidence of congenital or acquired heart disease, and (c) normal findings by 2D and Doppler echocardiography, with no structural abnormality and with normal chamber dimensions and systolic performance. Exclusion criteria were (a) structural congenital or acquired heart disease, (b) abnormal cardiac rhythms, (c) subjects who have other systemic diseases affecting LV function, and (d) subjects above 6 years of age. Demographic data, as age and gender, were collected during the performance of the echocardiographic studies. Approval of X University ethical committee was taken (MS 157/2021, FWA 000017585), and informed consent was obtained from the subjects’ guardians.

### Two-dimensional transthoracic echocardiography

Study subjects underwent a transthoracic echocardiography (TTE) examination guided by the echocardiography recommendations of the American Society [7, 8]. Every examination was performed at rest. In agitated infants and children, sedation with chloral hydrate aqueous solution (50 mg per kilogram) 15 min prior to the study was done. Images were obtained with a 4.5 MHz (M5S) or a 8 MHz (6S) matrix transducer using a commercially available system, the Vivid E9 echocardiographic scanner (GE Ultrasound, Horten, Norway).

Complete 2D, Doppler, and color Doppler were performed in all accessible windows including parasternal long-axis; parasternal short-axis (basal, mid, and great vessels); apical four-chamber, two-chamber, five-chamber, and three-chamber; subcostal; and suprasternal views with ECG physio signal displayed with all detected echo-Doppler study with loop recording of two to three cycles. All images were stored for the offline analysis using the EchoPAC GE 201 version (Chicago, Illinois, USA).

### Routine echocardiographic parameters

Quantifications of cardiac chamber size, ventricular mass, and systolic and diastolic LV function were done in accordance with the recommendations for chamber quantification of the American Society of Echocardiography [7, 8]. Routine 2D echocardiographic images were acquired in the various views as parasternal and apical. The LV end-diastolic (ED) dimension and end systolic (ES), fractional shortening (FS), EF and left atrial (LA) dimension were obtained in the parasternal short-axis view. TDI was done at the interventricular septum base (septal data), at the LV lateral wall (lateral data). Gain was optimized to obtain clear signals, and images were recorded, so myocardial velocity during systole (s’) was assessed [7, 8]. The tricuspid annular plane systolic excursion (TAPSE) was measured by 2D echocardiography-guided M-mode recordings from the apical four-chamber view as previously recommended [7, 8].

### Two-dimensional speckle tracking echocardiography

Two-dimensional B-mode multiframe (grayscale) images were acquired in the apical four-, two-, three-chamber views and parasternal short-axis view at the papillary muscle (PM) level and parasternal short-axis view at the mitral valve (MV) level. Cine loops were used to store data for the offline analysis. The images were analyzed offline using customized software (EchoPAC V113; GE Healthcare). The LV endocardial boundary was manually delineated; the software automatically drew the LV epicardial boundary. Manual adjustment of the width of the region of interest was done when needed. Then, the software divided the LV myocardium into six segments and generates global and segmental longitudinal, circumferential, and radial strains. Because the myocardium is shortened during systole in the longitudinal direction, the longitudinal strain appears below the baseline.

From these curves, peak systolic longitudinal, circumferential, and radial strains were recorded for each of the myocardial segments. The strain values for all the segments are recorded and averaged to obtain the global longitudinal strain (GLS), global circumferential strain (GCS), and global radial strains (GRS). Strain values are presented as percentages. Negative strain values reflect shortening, whereas positive strain values reflect lengthening or thickening [8].

### Three-dimensional speckle tracking echocardiography

**Fig. 1:**
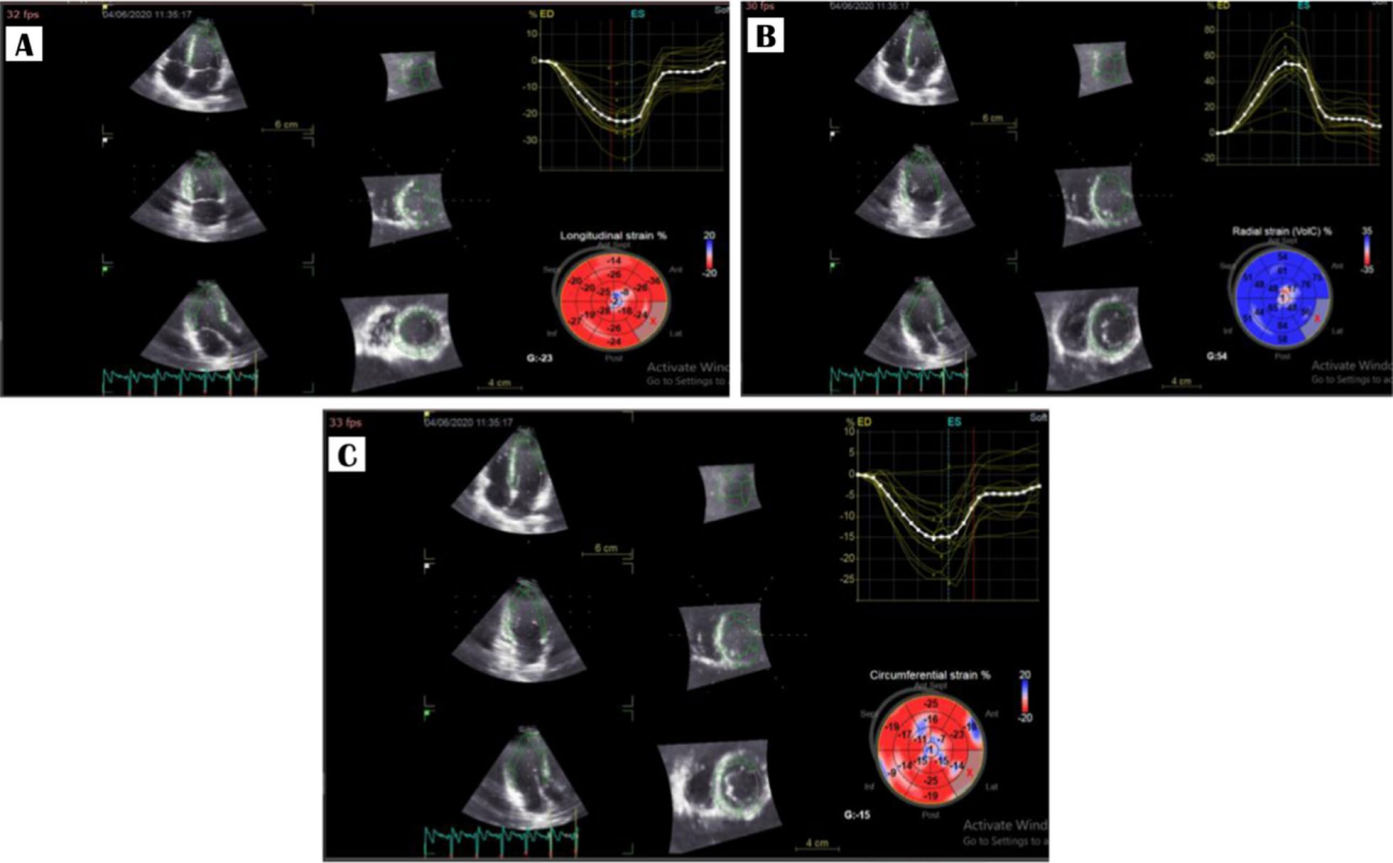
Three-dimensional (3D) tracing of the left ventricle (LV) to estimate **A** global longitudinal strain (GLS). **B** Global radial strain (GRS). **C** Global circumferential strain (GCS)

Commercially available ultrasound system (Vivid E95, GE Healthcare, Milwaukee WI) was used for data acquisition and using a phased-array matrix transducer. Studies were performed by experienced cardiologist with subjects in the supine position. From the apical position, full-volume data sets were acquired. To ensure the acquiring of the full LV volume within the pyramidal scan volume, multiple cardiac cycles data sets were acquired using wide angle, through which four wedge-shaped sub-volumes were acquired with ECG gating. The mean frame rate of RT3DE data sets was 20.7 6 frames/sec. RT3DE data sets were analyzed using 3D LV analysis software (EchoPac) by an experienced cardiologist. From the 3D full-volume data sets, the apical four-chamber view was automatically extracted. Non-foreshortened apical view was identified by finding in the data set views with the largest LV long-axis dimensions from the apex and the MV. LV boundaries were selected by manually selecting the annulus of the MV and the apex of the LV, after which automatic reconstruction of the 3D endocardial surface was achieved, and when needed manual adjustments of the surface of the endocardium were applied. Subsequently, 3D STE analysis was automatically performed throughout the cardiac cycle. Using the known segmentation scheme, the LV was then automatically divided into 16 segments. The software provided segmental longitudinal, circumferential, radial curves, from which peak global strain and averaged peak strain at the basal, midventricular and apical LV levels were determined. 3D strain shows the tangential deformation and is calculated using the sum of the longitudinal and circumferential strain components, without including the radial component. Anatomically, it may present the result of contraction direction of the muscle fibers that are aligned to the myocardial surface. In case there was poor-quality 3D images, subjects were excluded [8]. After analysis, the final recorded data will include LVED volume, LVES volume, EF, LV mass, stroke volume (SV), and cardiac output (COP) together with GLS, GCS, and GRS (Fig. 1).

### Reproducibility

Ten subjects were randomly selected to assess inter- and intra-observer agreement of 3D strain analysis for GLS, GCS, and GRS blinded to the previous results and using new arbitrary images. For the inter-observer variability assessment, the first operator performed the analyses. The second operator repeated the analyses within 24 h. For intra-observer variability assessment, the analyses were repeated two times by the first operator within 1 week.

### Statistical analysis

Results were analyzed using the Statistical Package for the Social Sciences Software (SPSS version 25.0; IBM corp., Armonk, New York, USA). Continuous variables were expressed as the mean ± SD, whereas categorical variables were expressed as frequencies and percent. The associations between variables were assessed by Pearson’s r correlation analysis. *P* less than or equal to 0.05 was accepted as statistically significant with confidence interval (CI) greater than 95%, and *P* less than or equal to 0.001 was considered highly significant; the relations between age and 2D and 3D global strain (GS) parameters were determined using scatterplots, one-way analysis of variance, and second-order polynomial regression analysis and linear regression analysis.

## Results

### Two-dimensional echocardiography

**Fig. 2:**
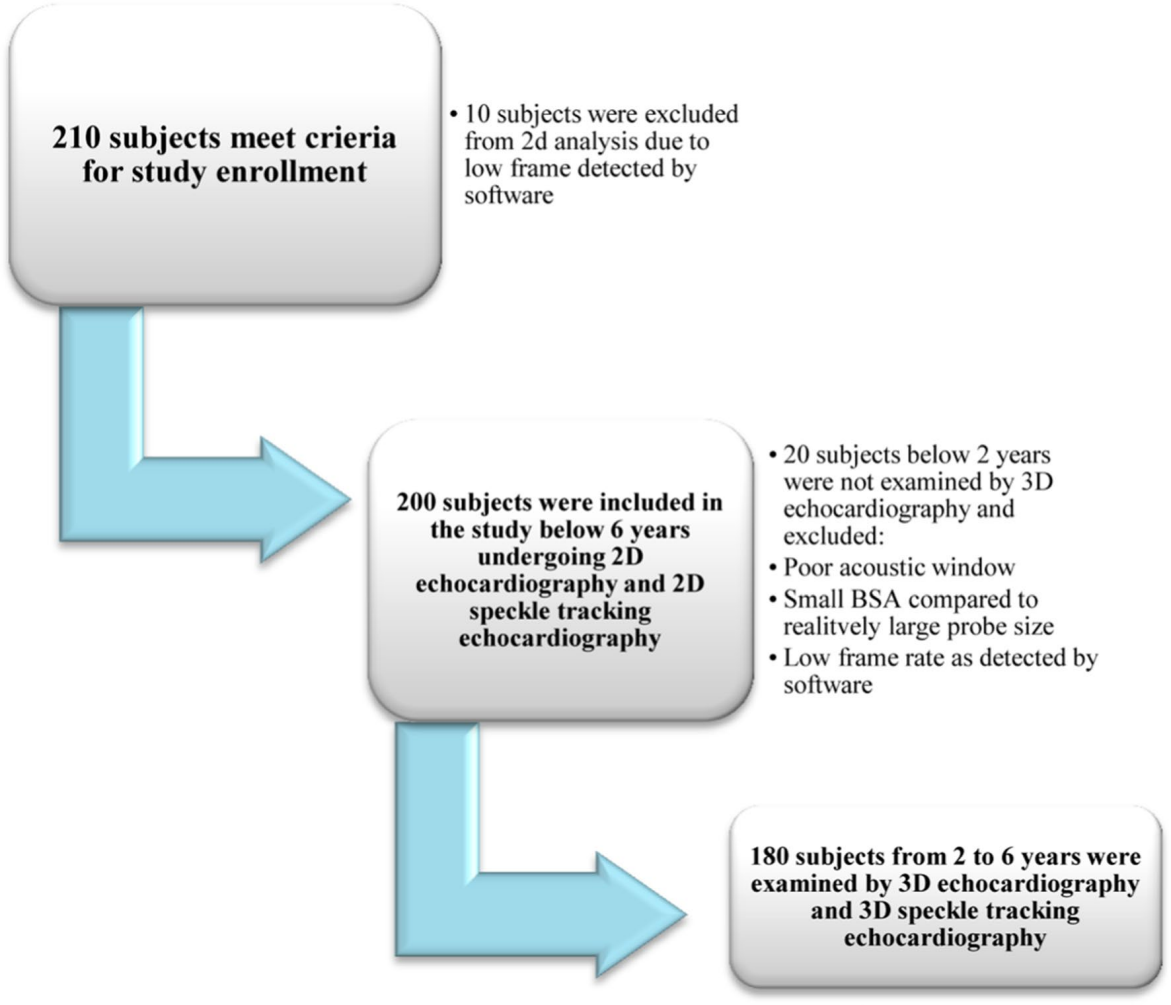
Diagram of subjects examined by two-dimensional (2D) and three-dimensional echocardiography

Due to low frame rate detected by the software, 10 subjects were excluded from the beginning of the study from the 2D analysis, so the study involved 200 subjects (95%) below 6 years to assess the LV systolic function among healthy Egyptian preschool-aged pediatric children using conventional 2D echocardiography, 2D and 3D STE (Fig. 2). One hundred and four were males representing 52% of the study subjects. Demographic data are represented in Table 1.

**Table 1:** Study population demographic data and study population two-dimensional guided M-mode echocardiographic parameters

	Range			Mean	±	SD
*Demographic data*						
Age (years)	0.1	–	5.9	3.832	±	1.522
Weight (KG)	3.5	–	28	17.145	±	4.901
Height (M)	0.11	–	1.24	1.012	±	0.178
BSA (m2)	0.22	–	1.1	0.695	±	0.151
*Study population 2D echocardiography parameters(n* = *200)*						
AO (mm)	11	–	21	16.610	±	2.088
LA (mm)	13	–	29	22.810	±	3.275
IVSd (mm)	4	–	9	4.970	±	1.147
IVSs (mm)	4	–	9	7.320	±	1.031
LVPWd (mm)	3	–	8	4.880	±	1.025
LVPWs (mm)	5	–	10	7.470	±	1.129
LVIDd (mm)	18	–	49	36.870	±	6.601
LVIDs (mm)	11	–	33	23.330	±	5.871
EF %	55	–	78	64.400	±	4.592
FS%	27	–	45	32.720	±	3.170
TAPSE (mm)	18	–	28	22.220	±	2.503
S’				11.1	±	2.10

*AO* aortic root, *LA* left atrium, *BSA* body surface area, *IVSd* interventricular septum in diastole, *IVSs* interventricular septum in systole, *LVPWd* left ventricular posterior wall in diastole, *LVPWs* left ventricular posterior wall in systole, *LVIDd* left ventricular internal dimension in diastole, *LVIDs* left ventricular internal dimension in systole, *EF* ejection fraction, *FS* fractional shortening, *S*’ myocardial velocity during systole, *TASPSE* tricuspid annulus plane systolic excursion

Conventional echocardiographic parameters of the study subjects were assessed as S’ mean + _ SD which was 11.1 + _2.1. Other echocardiographic parameters are presented in Table 1.

#### Two-dimensional speckle tracking echocardiography

Regarding the 2D STE parameters: our study population GLS ranged from -18.1% to -30.7% with a mean of -22.1345 ± 2.166%. GCS was from -16.7% to -23.4% with a mean of -19.02 ± 1.23%. And GRS ranged from 37.4% to 52.6% with a mean of 42.25 ± 2.35%. Data were related and categorized according to age (Table 2). Also, linear correlation analysis was made, and data were categorized according to age.

**Table 2:** Mean of 2D speckle tracking echocardiography (STE) parameter in subjects of our study categorized according to age-group and *r* and *P* values of correlation between 2D strain and age

	< 1 year	> 1 ≤ 2 years	> 2 ≤ 3 years	> 3 ≤ 4 years	> 4 ≤ 5 years	> 5 ≤ 6 years	Total	*r*	*P*
No	12	12	39	43	41	53	200		
2D GLS%	− 23.291 ± 7.314	− 24.491 ± 1.105	− 23.555 ± 0.595	− 22.338 ± 0.745	− 21.140 ± 0.670	− 19.957 ± 0.882	− 22.1345 ± 2.166	0.898	0.001
2D GCS%	− 18.95 ± 0.922	− 19.1 ± 0.7508	− 19.447 ± 1.341	− 18.704 ± 1.221	− 18.941 ± 0.894	− 19.035 ± 1.498	− 19.021 ± 1.238	0.077	0.28
2D GRS%	48.433 ± 2.7002	44.583 ± 1.151	43.413 ± 0.864	42.445 ± 0.825	41.307 ± 0.798	40.038 ± 1.054	42.25 ± 2.35	− 0.886	0.001

*2D* two-dimensional, *GLS* global longitudinal strain, *GCS* global circumferential strain, *GRS* global radial strain

**Fig. 3:**
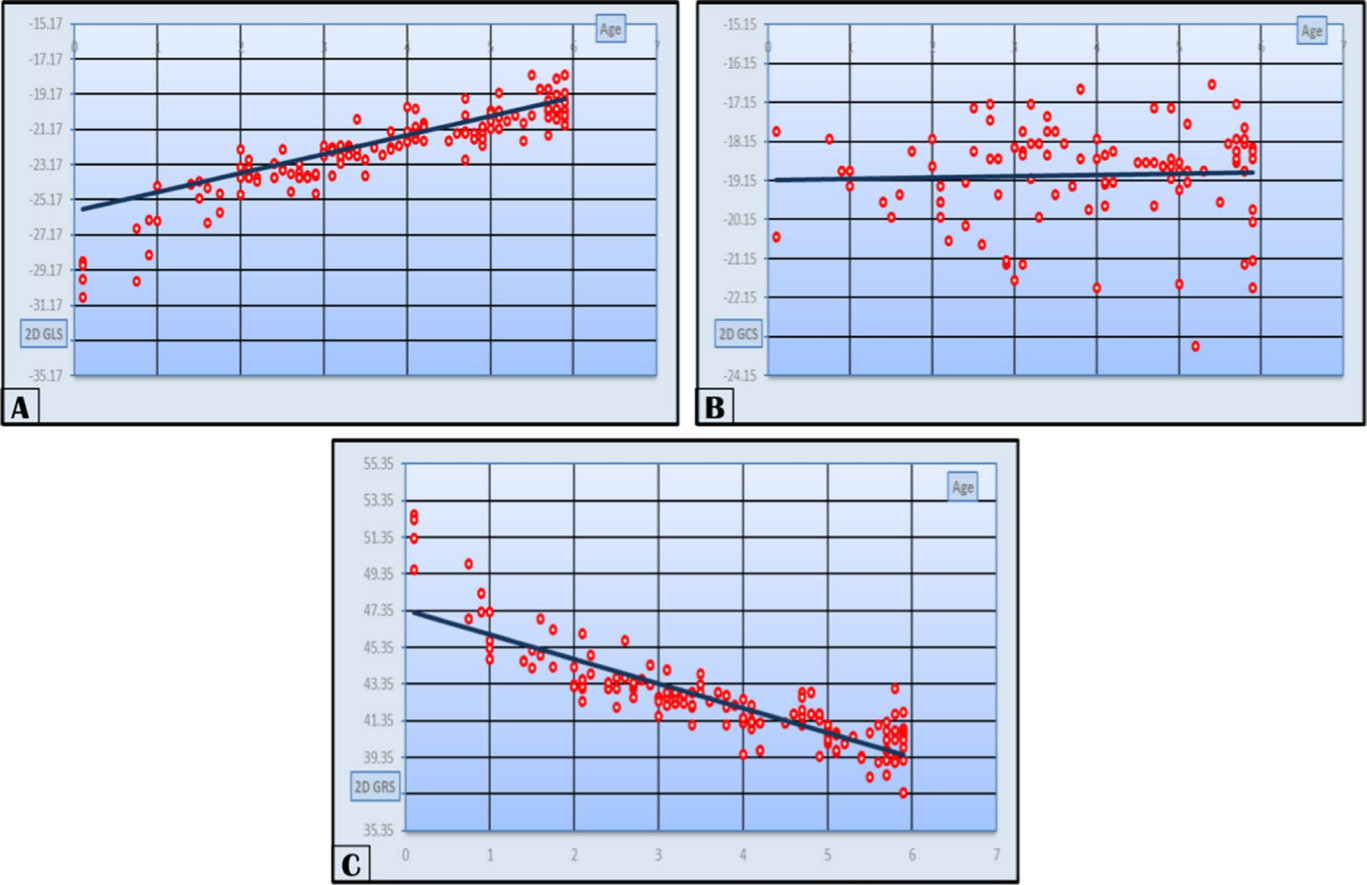
Our study population 2D **A** GLS STE in relation to age, **B** GCS STE in relation to age, **C** GRS STE in relation to age

There was a strongly positive significant correlation between age and 2D values of GLS. The GCS showed a weakly positive nonsignificant correlation with age, while GRS showed a strongly negative significant correlation with age as shown in Table 2 (Fig. 3).

### Three-dimensional echocardiography parameters

#### Feasibility

The 3D echocardiographic parameters were performed only to subjects from 2 to 6 years with a total of 180 subjects (85%), while subjects less than 2 years (20 subjects) were excluded due to poor acoustic window and small BSA compared to relatively large probe size, leading to inability to acquire proper image for analysis or low frame rate as detected by the software (Fig. 2). The 3D echocardiographic parameters of the study subjects are presented in Table 3.

**Table 3:** Our study population 3D echocardiographic parameters

3D echocardiography parameters						
*n* = 180	From 2 to 6 years					
	Range			Mean	±	SD
EDV (ml)	27	–	86	51.58	±	12.028
Indexed EDV (ml/m2)	28.2	–	148.2	73.607	±	21.817
ESV (ml)	10	–	37	23.367	±	6.709
Indexed ESV (ml/m2)	11	–	112.1	34.032	±	13.680
EF %	55	–	73	62.622	±	3.511
SV (ml)	32	–	52	41.100	±	4.067
COP (L/min)	3.1	–	43	4.627	±	4.104
ED mass (g)	41	–	86	63.767	±	9.042
Indexed ED mass (g/m2)	47.3	–	144.2	90.404	±	20.297
ES mass (g)	43	–	87	66.167	±	9.226
Indexed ES mass (g/m2)	52.5	–	148	93.906	±	20.734

*EDV* end-diastolic volume, *BSA* body surface area, *ESV* end-systolic volume, *EF* ejection fraction, *SV* stroke volume, *COP* cardiac output, *ED mass* end diastolic mass, *ES mass* end systolic mass

#### Three-dimensional speckle tracking echocardiography

**Fig. 4:**
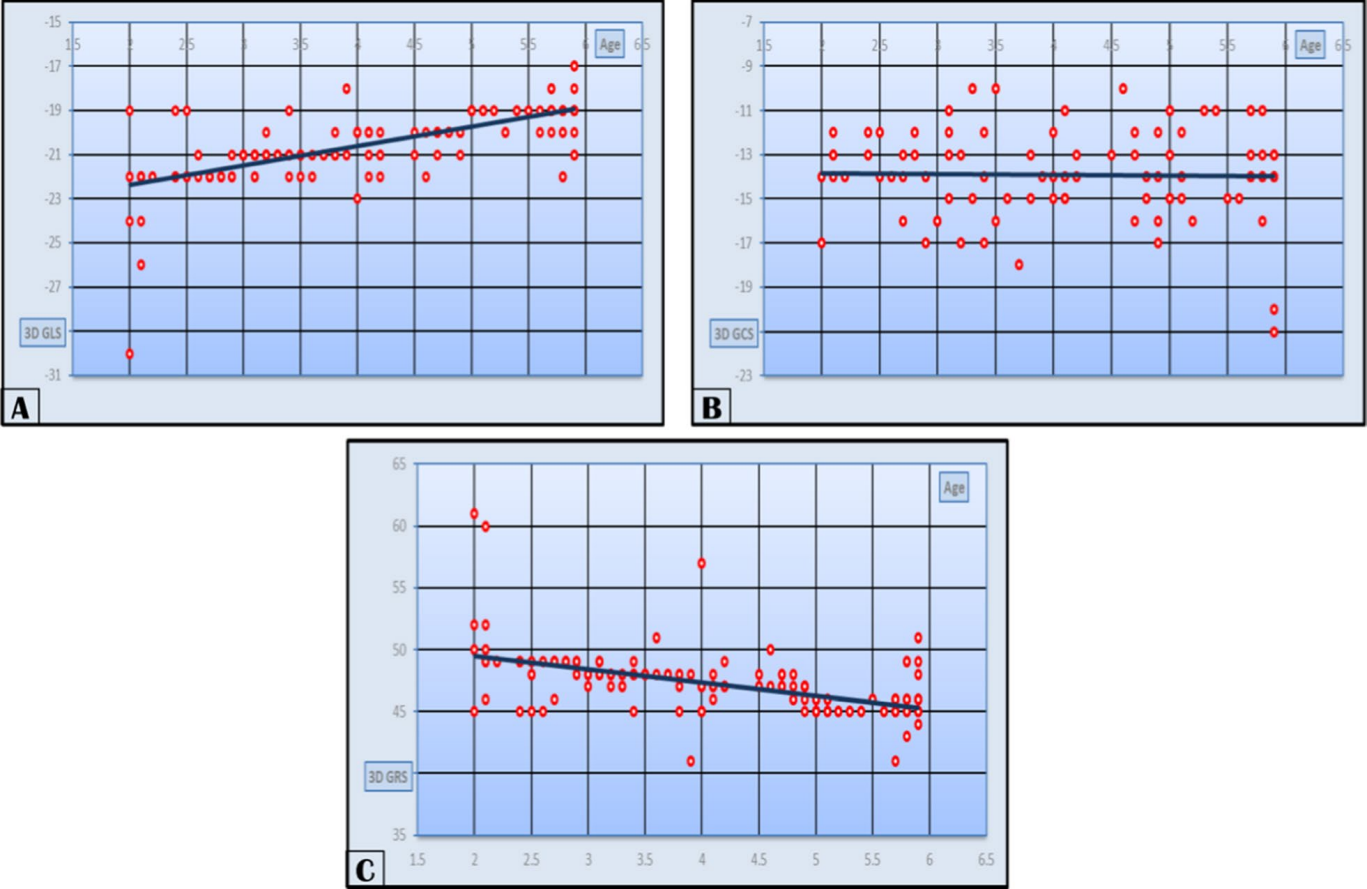
Our study population 3D STE **A** GLS STE in relation to age, **B** GCS STE in relation to age, **C** GRS STE in relation to age

Regarding the 3D STE data, GLS ranged from -17% to -30% with a mean of -20.48 ± 1.526%. GCS ranged from -10% to -21% with a mean of -13.90 ± 2.05%, while GRS ranged from 41 to 61% with a mean of 47.21 ± 2.382%. Data were related and categorized according to age as in Table 4. Also, linear correlation analysis was made, and data were categorized according to age. 3D GLS values had a strongly positive significant correlation with age, while GCS showed a weakly positive nonsignificant correlation and GRS showed a strongly negative significant correlation as shown in Table 4 (Fig. 4).

**Table 4:** Mean of 3D speckle tracking parameter in subjects of our study categorized according to age-group and *r* and *P* values of correlation between 3D strain and age

	< 1 < 2 year	≥ 2 ≤ 3 years	> 3 ≤ 4 years	> 4 ≤ 5 years	> 5 ≤ 6 years	Total	*r*	*P*
No	NA	43	43	41	53	180		
3D GLS%	NA	− 22.79 ± 2.862	− 20.89 ± 0.813	− 20.1 ± 0.79	− 19.19 ± 0.908	− 20.48 ± 1.526	0.766	0.001
3D GCS%	NA	− 14.645 ± 1.612	− 14 ± 2.241	− 13.71 ± 1.798	− 13.92 ± 2.424	− 13.9 ± 2.05	0.055	0.955
3D GRS%	NA	50.405 ± 4.515	47.77 ± 2.067	46.79 ± 1.071	45.6 ± 1.485	47.21 ± 2.382	− 0.653	0.001

*3D* three-dimensional, *GLS* global longitudinal strain, *GCS* global circumferential strain, *GRS* global radial strain

### Correlation between two-dimensional and three-dimensional speckle tracking echocardiography

**Fig. 5:**
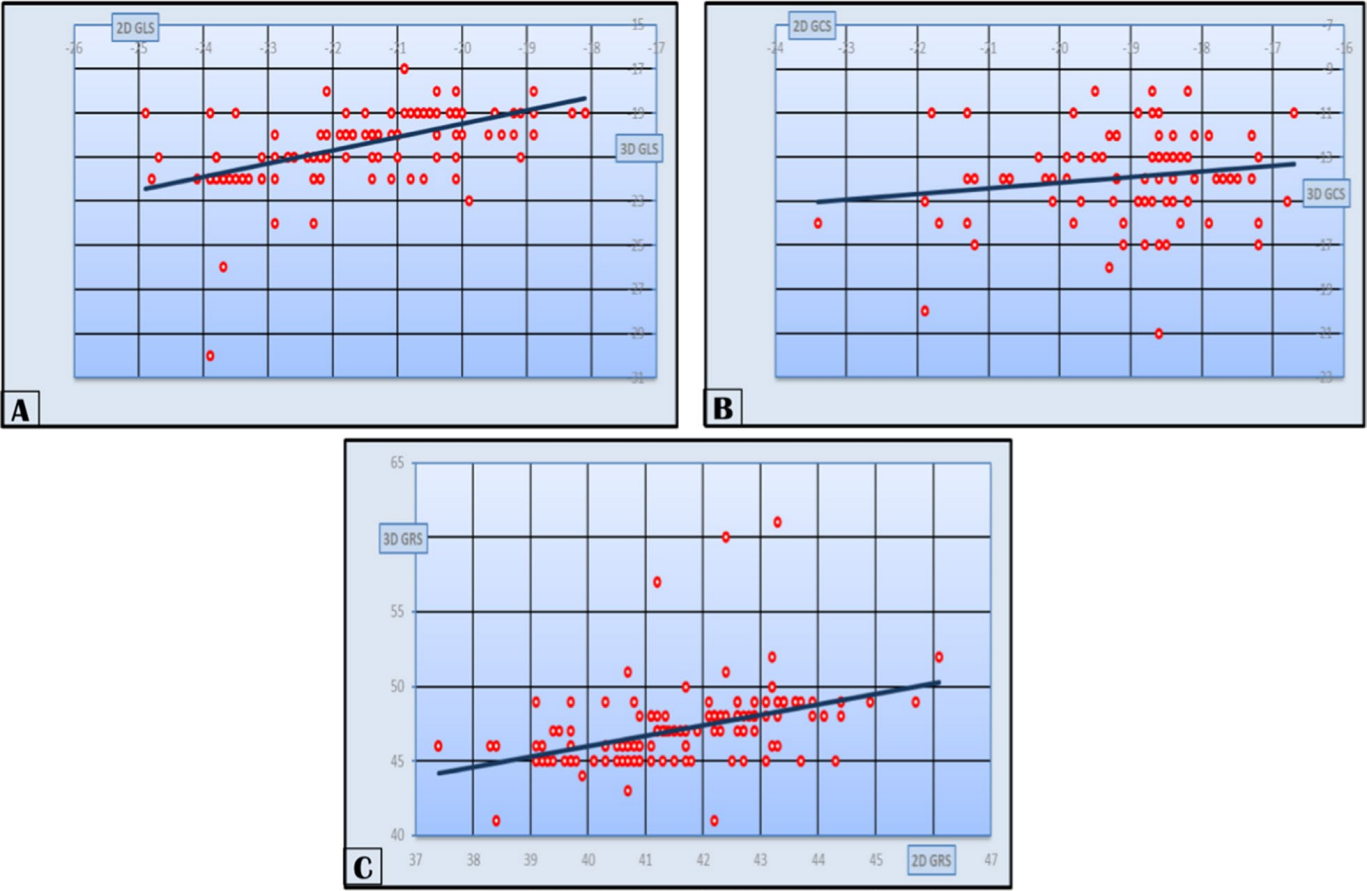
Linear correlation between **A** 2D and 3D GLS, **B** 2D and 3D GCS, **C** 2D and 3D GRS

Linear correlation analysis of 2D and 3D values of strain showed that GLS had a strongly positive significant correlation, while GCS showed a weakly positive nonsignificant correlation and GRS showed a strongly positive significant correlation as shown in Table 5 (Fig. 5).

**Table 5:** *r* and *P* values of correlation among our study population 2D and 3D values of strain

	*r*	*P*
2D-3D GLS	0.702	0.001
2D-3D GCS	0.105	0.161
2D-3D GRS	0.6	0.001

*2D* two-dimensional, *3D* three-dimensional, *GLS* global longitudinal strain, *GCS* global circumferential strain, *GRS* global radial strain

## Discussion

Reference value studies for myocardial deformation parameters in children are relatively few, especially in the preschool-aged children. In this single-center study, we aimed to determine normal range values for LV systolic function among healthy Egyptian preschool pediatric population using 2D and 3D STE. It is one of the largest reports to date of 2D and 3D STE in preschool-age children with structurally normal hearts and no cardiovascular disease.

### Normal range and age correlation in left ventricle two-dimensional strain

In our study, 2D STE GLS strain ranged from -18.1% to -30.7% with a mean of –22.1345 ± 2.166%, GCS was from -16.7% to -23.4% with a mean of -19.02 ± 1.23%.,while GRS ranged from 37.4% to 52.6% with a mean of 42.25 ± 2.35%.

From the previous reports that studied all strain measurements and correlated with our values with slight differences, the study conducted by Liselotte M. Klitsie et al. included 37 children in the study group below 1 year of age. The mean strain values were -20.6 ± 3.1% for GLS , -21.7 ± 4.7 for GCS, and 40.5 ± 13.5 for GRS in the study group of 1–4 years. Their mean strain values were 23.6 ± 1.5% for GLS, -22.1 ± 3.6% for GCS, and 49.8 ± 13.7% for GRS. All data were collected and analyzed using GE VIVID 7 echo system with EchoPAC 11.1.8 [9].

Several previous studies did not include radial strain and were conducted by different echo vendor machines as the following studies that used the Philips echo machine. Adi Adar et al. conducted a study with subjects from 1 to 5 years where the GLS mean was -25.22 ± 2.43% and GCS mean was -23.89 ± 3.72%. All data were collected and analyzed using Philips EPIQ system [10]. Massimiliano Cantinotti et al. conducted a study with age-group below 2 years where the GLS mean was -26 ± 2.3% and GCS mean was -24.6 ± 4.2%. Also, in his study with age-group from 2 to 5yrs, the mean GLS was -25 ± 2.2% and GCS mean was -23.3 ± 4.3%. Imaging and analysis were done using Philips iE33 system [11]. Also, Laurens P. Koopman et al. conducted a study in the age-group from 1 to 6 years, where the GLS mean was -22.3 ± 2.2%, while the GCS mean was -23.8 ± 2.6%. All data were collected and analyzed using Philips iE33 medical system of echocardiography [2].

While other studies had differences as regard measurements, especially the GCS, Shaimaa et al. conducted a study with an older age-group ranging from 6 to 9 years. Data were collected using GE VIVID E9 echocardiography system. They found that GLS mean in the whole study population was -22.5 ± 2.56%, while GCS mean value was -33.9 ± 8.59% and GRS mean value was 56.5 ± 9.52% [12].

On categorizing our study population into age-groups and correlating the data with age-groups, we found that circumferential systolic strain of the left ventricle is independent of age, while longitudinal strain of the left ventricle decreases with increasing age. And also, there was a correlation of radial strain with age. There was a strong positive significant correlation between age and 2D values of strain with *r* value in GLS of 0.913 and *P* value of 0.001. The GCS showed a weakly positive nonsignificant correlation, *r* value was 0.074 and *P* value was 0.475, while GRS showed a strongly negative significant correlation, with *r* value of -0.86 and *P* value of 0.001.

Several studies have reported reduced longitudinal strain with increasing age, in agreement with our results. Yet other previous studies have reported that GLS is not age dependent. These discrepant results might partly be related to the different modalities, the software used for analysis, and the size of the study populations [4].

We found that circumferential systolic strain of the left ventricle is independent of age, while longitudinal strain of the left ventricle decreases with increasing age. And also, there was a correlation with radial strain with age. There was a strong positive significant correlation between age and 2D values of strain with *r* value in GLS of 0.913 and *P* value of 0.001. The GCS showed a weakly positive nonsignificant correlation, *r* value was 0.074, and *P* value was 0.475, while GRS showed a strongly negative significant correlation, with *r* value of -0.86 and *P* value of 0.001.

In Adi Adar et al. study, data were correlated with age and they found GLS has a weak positive significant correlation with age with *r* value of 0.34 and *P* value less than 0.001. But GCS was found to have a nonsignificant correlation with age with *P* value 0.07 which was similar to our study [10]. Also, Laurens P. Koopman et al. study data were correlated with age and they stated a weakly positive but significant correlation between GLS and age with *r* value of 0.33 and *P* value of 0.001, while GCS correlation with age was weak and nonsignificant with *r* value 0.03 and *P* value 0.79 [2]. It was in agreement too with our study with differences regarding GLS which showed a strong positive correlation which can be explained by our larger study group number and different vendor machine used. Also, as regard to correlation with age Massimiliano Cantinotti et al., in their study that included 239 healthy children up to 5 years of age, stated that there was a weak correlation between strain values and age with *r* value less than 0.01 and did not include radial strain, yet this study was done using the Philips iE3 [11], while other studies despite using the GE vivid echo vendor machine as Liselotte M. Klitsie et al. regarding correlation with age stated that all data were nonsignificant which can be explained by a small number of subject group and different ethnicity [9].

### Normal range and age correlation in left ventricle three-dimensional strain

Three-dimensional STE is a new technology used to assess LV global and regional function. 3D STE supposed to be more precise than 2D STE, because the speckles are within the pyramidal volume scan. So, 3D STE should remove the confounding effects of through-plane myocardial motion, which may affect the preciseness of LV strain measurements using 2D STE. Yet, the low temporal and spatial resolution of 3DE could adversely affect the preciseness of 3D STE measurements in patients with sets at the low frame rate range [4].

The quality of the image remains an important factor in the ability to acquire accurate stress and strain data by both 2D and 3D methods. However, the low frame rates that inevitably come with 3D imaging cannot be overcome yet. Another potential source of error is the failure to acquire the entirety of the LV apex when the 3D dataset is obtained from the apical four-chamber window [13].

Three-dimensional LV speckle studies were done on young age-group as our study is very scarce. Also, in our study young age-group below 2 years were excluded which were 20 subjects due to inadequate image quality. So, we finally included only 180 children aged 2–6yrs that underwent 3D STE.

Regarding the 3D STE data of our study, GLS ranged from -17% to −30% with a mean of -20.73 ± 2.03%. GCS ranged from -10% to -21% with a mean of 13.90 ± 2.05%, while GRS ranged from 41 to 61% with a mean of 47.51 ± 4.82%.

From the previous reports that studied all strain measurements, Zhou Lin et al. study included 51 children with a mean age 11.3 ± 2.1 years; their 3D GLS was 19.84 ± 2.73%, GCS was -19 ± 3.38% and GRS was 53.73 ± 11.17%. GE vivid E 95 was used to collect and analyze the data [14]. Also, Kyoko et al. concluded a study that included 15 subjects below 3 years of age with mean age 2 ± 1 year, with their GLS mean of -22.7 ± 2.6%, GCS mean of -29.3 ± 3% and GRS mean of 88.6 ± 6.1%, and another group of age ranged from 4 to 9 years with a mean age of 7 ± 2 years, their GLS mean of -21.9 ± 2.1%, GCS mean of -29.4 ± 4.3% and GRS mean of 92.7 ± 20.8%. All the data and analysis were done using Philips IE33 system [4]. While Perixu Zhang et al.’s study included 195 children with a mean age of 9.56 ± 1.21, their 3D strain values were GLS -19.54 ± 2.15%, GCS -23.14 ± 3.48%, and GRS 58.14 ± 11.59%. Toshiba echo system was used to collect and analyze the data [15]. All studies were in agreement with our results as regard to GLS, yet differences in GRS and GCS were evident which may be attributed to different vendor machines used as previous studies reported findings of lower circumferential strain values using the General Electric platform compared to the Philips platform and highlights the need for vendor-dependent normal values for circumferential strain until standardization initiatives have been deployed for this particular deformation parameter [2]. Also, there may be ethnicity difference and study group ages, BSA, and study population number discrepancies.

LV architecture can be reflected by the difference in the movement of the longitudinal versus the circumferential fibers, where both fibers are not interspersed but, on the contrary, are thought to be distinct regionally. That is, the fibers of the mid-wall are predominantly circumferential, whereas those of both the sub-endocardium and sub-epicardium are mostly longitudinal. Before reports of maturational changes of strain have been conflicting, with some reports showing no association and others describing relations of varying strength. These differences may be related to vendor-based differences in measurement or analysis, or to the specific age range in each age-group [10].

Stratifying this deformation parameter for age is needed. The short duration of each cardiac cycle may reflect the high strain rate values in comparison with older children. Strain values are usually comparable in different age-groups, so when the cardiac cycle is short, the deformation rate should be higher. Since heart rate and age show a very strong collinearity, this hypothesis is difficult to prove [2].

In our study, 3D GLS values had a strongly positive significant correlation with age with *r* value of 0.766 and *P* value of 0.001, while GCS showed a weakly positive nonsignificant correlation with *r* value of 0.055 and *P* value of 0.955 and GRS showed a strongly negative significant correlation with *r* value of -0.653 and *P* value of 0.001.

From the previous studies that correlated all 3D strain measurements with age, Li Zhang et al. study included 106 children aged from 1 to 5 years that concluded with regard to correlation with age; a weak nonsignificant correlation between strain values and age with *r* values of GLS was 0.049, GCS 0.013 and GRS 0.012, and *P* values for all of them were more than 0.05. Yet all data were collected and analyzed using Philips iE33 echo system with an older software version used [16]. On the other hand, recently Joseph D. Kuebler et al. concluded a study that included 35 healthy children from 1- to 5 years and concluded with regard to correlation with age; there was a weak but significant correlation between GLS and age with *r* value 0.22 and *P* value 0.001. But GCS had a weak nonsignificant correlation with age with *r* value 0.09 and *P* value 0.15 which agreed with our study results. GRS was not included. All data were collected and analyzed using Philips IE33 system [13].

### Correlation between two-dimensional and three-dimensional left ventricle speckle strain values

In our study, correlation analysis of 2D and 3D values of strain showed that GLS had a strongly positive significant correlation with *r* value 0.702 and *P* value 0.001, while GCS showed a weakly positive nonsignificant correlation with *r* value 0.105 and *P* value 0.161 and GRS showed a strongly positive significant correlation with *r* value 0.6 and *P* value 0.001.

On the other hand, Sowmya et al. made a comparison between results of 2D and 3D STE with 17 subjects involved with a mean age of 13.9 years using Siemens medical system for data collection and analysis and they found that there was a positive strong significant correlation regarding GLS with *r* value 0.68 and *P* value 0.001. GCS showed a strong positive correlation with *r* value 0.62 and *P* value 0.004. GRS strain showed a weak nonsignificant correlation. All data were collected and analyzed using Siemens echo system [17]. In comparison with our study with Sowmya et al., there was concordance with our study with GLS, but GCS and GRS showed discordance with our study that may be attributed to different echo system used, different age-group, and different ethnicity.

Doaa Aly et al. recently conducted a study on 105 pediatric subjects with age from 1 to 18 years with normal cardiac structure correlating 2D and 3D GLS only, yet showed good agreement between both and correlation which was high with *r* value 0.7 and *P* value < 0.05; the analyses were performed using TOMTEC software. This shows that at least 3D GLS can be a promising good alternative to 2D GLS in assessing the LV function due to its less time consumption and less sophisticated analysis method [18].

### Limitations

Image quality remains a significant factor in the ability to obtain accurate stress and strain parameters by both 2D and 3D methods. However, the lower frame rates that inevitably accompany 3D imaging cannot yet be overcome. All subjects involved in the study had conventional and 2D STE, but not all of them had a 3D examination due to poor echogenic windows and relatively small BSA in comparison with large probe size and low frame rate as detected by the software. This study was constructed during the COVID-19 era in a single-center study which led to limited number of patients. Although this reference value study included around 200 participants, estimation of the mean values would have been more robust if more participants were included. Especially in younger children where the number of participants was lower, the normal values should be interpreted with caution.

## Conclusions

Three-dimensional global strain analysis using the 3D STE is feasible in the preschool-age pediatric population. The study involved 200 healthy Egyptian preschool-age pediatric subjects to assess for the normal range of 2D and 3D STE which is not settled yet and showed a lot of different reference ranges based on age and ethnicity of subjects involved.

We reported echocardiographic normal values for LV strain by 2D and 3D STE in healthy Egyptian children by using vendor-specific (Vivid E 95) software. Our results were almost concordant with previous observations in most of the values except for circumferential strain which showed discordance with previous observation, especially 3D values and 3D radial strain values which could be attributed to different vendor system used and different ethnicity. Further studies are required to reinforce these data.

## Data Availability

The datasets used and analyzed during the current study are available from the corresponding author on reasonable request.

## Abbreviations

BSA: Body surface area
COP: Cardiac output
CI: Confidence interval
EF: Ejection fraction
ED: End-diastolic
ES: End-systolic
FS: Fractional shortening
GCS: Global circumferential strain
GLS: Global longitudinal strain
GRS: Global radial strain
GS: Global strain
LA: Left atrium
LV: Left ventricle
M-mode: Motion mode
MV: Mitral valve
PM: Papillary muscle
PW: Pulse wave
RT3DE: Real-time three-dimensional echocardiography
STE: Speckle tracking echocardiography
SV: Stroke volume
TAPSE: Tricuspid annular plane systolic excursion
TDI: Tissue Doppler imaging
TTE: Trans-thoracic echocardiography
2D: Two-dimensional
3D: Three-dimensional
3DE: Three-dimensional echocardiography

## References

[1] Galderisi M (2011). Diagnosis and management of left ventricular diastolic dysfunction in the hypertensive patient. Am J Hypertens.

[2] Koopman LP, Rebel B, Gnanam D (2019). Reference values for two- dimensional myocardial strain echocardiography of the left ventricle in healthy children. Cardiol Young.

[3] Jashari H, Rydberg A, Ibrahimi P (2015). Normal ranges of left ventricular strain in children: a meta-analysis. Cardiovasc Ultrasound.

[4] Kaku K, Takeuchi M, Tsang W, Takigiku K, Yasukochi S, Patel AR, Mor-Avi V, Lang RM, Otsuji Y (2014). Age-related normal range of left ventricular strain and torsion using three-dimensional speckle-tracking echocardiography. J Am Soc Echocardiogr.

[5] Poutanen T, Jokinen E, Sairanen H (2003). Left atrial and left ventricular function in healthy children and young adults assessed by three-dimensional echocardiography. Heart.

[6] Nabeshima Y, Seo Y, Takeuchi M (2020). A review of current trends in three-dimensional analysis of left ventricular myocardial strain. Cardiovasc Ultrasound.

[7] Gardin JM, Adams DB, Douglas PS (2002). Recommendations for a standardized report for pediatric transthoracic echocardiography: a report from the American Society of Echocardiography's Nomenclature and Standards Committee and Task Force for a Standardized Echocardiography Report. J Am Soc Echocardiogr.

[8] Lang RM, Badano LP, Mor-Avi V, et al. the American society of echocardiography recommendations from cardiac chamber quantification in adults: a quick reference guide from ASE workflow and lab management task force. J Am Soc Echocardiogr 2018(31):1–20.

[9] Klitsie LM, Roest AA, van der Hulst AE, Stijnen T, Blom NA, Ten Harkel AD (2013). Assessment of intraventricular time differences in healthy children using two-dimensional speckle-tracking echocardiography. J Am Soc Echocardiogr.

[10] Adar A, Ghelani SJ, Sleeper LA, Lu M, Marcus E, Ferraro AM, Colan SD, Banka P, Powell AJ, Harrild DM (2019). Normal values for left ventricular strain and synchrony in children based on speckle tracking echocardiography. Am J Cardiol.

[11] Cantinotti M, Scalese M, Giordano R (2018). Normative data for left and right ventricular systolic strain in healthy Caucasian Italian children by two-dimensional speckle-tracking echocardiography. J Am Soc Echocardiogr.

[12] Habib SA, Ahmad GM, Mohamed LA, Mohamed RA (2019). Reference values for left ventricular strain using 2-dimensional speckle tracking in primary school-aged healthy Egyptian children. Al-Azhar Assiut Med J.

[13] Kuebler JD, Ghelani S, Williams DM (2018). Normal values and growth-related changes of left ventricular volumes, stress, and strain in healthy children measured by 3-dimensional echocardiography. Am J Cardiol.

[14] Lin Z, Zheng J, Chen W (2020). Assessing left ventricular systolic function in children with a history of Kawasaki disease. BMC Cardiovasc Disord.

[15] Zhang P, Li D, Yanzhuo Su (2018). Assessment of myocardial strain in children with risk factors for atherosclerosis with use of 3D speckle tracking echocardiography. Echocardiography.

[16] Zhang L, Gao J, Xie M, Yin P, Liu W, Li Y, Klas B, Sun J, Balluz R, Ge S (2013). Left ventricular three-dimensional global systolic strain by real-time three-dimensional speckle-tracking in children: feasibility, reproducibility, maturational changes, and normal ranges. J Am Soc Echocardiogr.

[17] Balasubramanian S, Punn R, Smith SN, Houle H, Tacy TA (2017). Left ventricular systolic myocardial deformation: a comparison of two-and three-dimensional echocardiography in children. J Am Soc Echocardiogr.

[18] Aly D, Kuzava L, Samrany A, Parthiban A (2020). Assessment of left ventricular systolic function by speckle tracking deformation imaging: a comparison between two- dimensional and three-dimensional echocardiography in healthy children. JACC.

